# Effects of pemafibrate on glucose metabolism markers and liver function tests in patients with hypertriglyceridemia: a pooled analysis of six phase 2 and phase 3 randomized double‐blind placebo‐controlled clinical trials

**DOI:** 10.1186/s12933-021-01291-w

**Published:** 2021-05-04

**Authors:** Koutaro Yokote, Shizuya Yamashita, Hidenori Arai, Eiichi Araki, Mitsunori Matsushita, Toshiaki Nojima, Hideki Suganami, Shun Ishibashi

**Affiliations:** 1 Department of Endocrinology, Hematology and Gerontology, Chiba University Graduate School of Medicine, 1-8-1 Inohana, Chuo-ku, Chiba-shi, Chiba, 260-8670 Japan; 2 Department of Diabetes, Metabolism and Endocrinology, Chiba University Hospital, 1-8-1 Inohana, Chuo-ku, Chiba-shi, Chiba, 260-8670 Japan; 3 Rinku General Medical Center, 2-23 Ohrai-kita, Rinku, Izumisano-shi, Osaka 598-8577 Japan; 4 National Center for Geriatrics and Gerontology, 7-430 Morioka-cho, Obu-shi, Aichi 474-8511 Japan; 5 Department of Metabolic Medicine, Faculty of Life Sciences, Kumamoto University, 1-1-1 Honjo, Chuo-ku, Kumamoto-shi, Kumamoto, 860-8556 Japan; 6 Medical Affairs Department, Kowa Company, Ltd, 3-4-14 Nihonbashi-honcho, Chuo-ku, Tokyo, 103-8433 Japan; 7 Clinical Data Science Department, Kowa Company, Ltd, 3-4-14 Nihonbashi-honcho, Chuo- ku, Tokyo, 103-8433 Japan; 8 Division of Endocrinology and Metabolism, Department of Internal Medicine, School of Medicine, Jichi Medical University, 3311-1 Yakushiji, Shimotsuke-shi, Tochigi 329-0498 Japan

**Keywords:** Pemafibrate, Glucose metabolism, Liver function, FGF21

## Abstract

**Background:**

Increased risk of cardiovascular events is associated not only with dyslipidemias, but also with abnormalities in glucose metabolism and liver function. This study uses pooled analysis to explore the in-depth effects of pemafibrate, a selective peroxisome proliferator-activated receptor α modulator (SPPARMα) already known to decrease elevated triglycerides, on glucose metabolism and liver function in patients with hypertriglyceridemia.

**Methods:**

We performed a post-hoc analysis of six phase 2 and phase 3 Japanese randomized double-blind placebo-controlled trials that examined the effects of daily pemafibrate 0.1 mg, 0.2 mg, and 0.4 mg on glucose metabolism markers and liver function tests (LFTs). Primary endpoints were changes in glucose metabolism markers and LFTs from baseline after 12 weeks of pemafibrate treatment. All adverse events and adverse drug reactions were recorded as safety endpoints.

**Results:**

The study population was 1253 patients randomized to placebo (n = 298) or pemafibrate 0.1 mg/day (n = 127), 0.2 mg/day (n = 584), or 0.4 mg/day (n = 244). Participant mean age was 54.3 years, 65.4 % had BMI ≥ 25 kg/m^2^, 35.8 % had type 2 diabetes, and 42.6 % had fatty liver. Fasting glucose, fasting insulin, and HOMA-IR decreased significantly in all pemafibrate groups compared to placebo. The greatest decrease was for pemafibrate 0.4 mg/day: least square (LS) mean change from baseline in fasting glucose − 0.25 mmol/L; fasting insulin − 3.31 µU/mL; HOMA-IR − 1.28. ALT, γ-GT, ALP, and total bilirubin decreased significantly at all pemafibrate doses vs. placebo, with the greatest decrease in the pemafibrate 0.4 mg/day group: LS mean change from baseline in ALT − 7.6 U/L; γ-GT − 37.3 U/L; ALP − 84.7 U/L; and total bilirubin − 2.27 µmol/L. Changes in HbA1c and AST did not differ significantly from placebo in any pemafibrate groups in the overall study population. The decreases from baseline in LFTs and glucose metabolism markers except for HbA1c were notable among patients with higher baseline values. FGF21 increased significantly in all pemafibrate groups compared to placebo, with the greatest increase in the pemafibrate 0.4 mg/day group. Adverse event rates were similar in all groups including placebo.

**Conclusions:**

In patients with hypertriglyceridemia, pemafibrate can improve glucose metabolism and liver function, and increase FGF21, without increasing adverse event risk.

**Supplementary Information:**

The online version contains supplementary material available at 10.1186/s12933-021-01291-w.

## Background

Dyslipidemia is frequently associated with type 2 diabetes (T2D) and with non-alcoholic fatty liver disease (NAFLD) [1, 2], and hypertriglyceridemia is a known risk factor for developing newly onset T2D and NAFLD [3, 4]. Cardiovascular events are common not only in patients with T2D and NAFLD [5, 6] but also even in patients with milder abnormalities of laboratory tests such as glucose metabolism and liver function [7, 8], suggesting that insulin resistance may contribute to increased risk of cardiovascular diseases. These findings emphasize the importance of identifying patients with abnormal laboratory results in glucose metabolism, liver function and plasma lipids as part of ongoing efforts toward cardiovascular disease prevention.

Peroxisome proliferator-activated receptor (PPAR) α agonists can reduce triglyceride (TG) and TG-rich lipoprotein cholesterol levels and improve atherogenic dyslipidemia [9]. Pemafibrate, also known as K-877, was developed as a selective PPARα modulator (SPPARMα), which provides a favorable benefit-risk ratio superior to that of other conventional PPARα agonists [10, 11]. The high specificity and selectivity of pemafibrate as SPPARMα are achieved by its Y-shaped molecular structure, which allows pemafibrate to bind optimally to the PPARα ligand binding domain [12, 13]. After binding to the PPARα, pemafibrate up-regulates the expression of specific genes involved in fatty acid metabolism, primarily in human hepatocytes [14]. Pemafibrate reduces TG by 45–51 %, while increasing high-density lipoprotein cholesterol (HDL-C) by 12–20 % [15]. Pemafibrate also decreases the value of liver function tests (LFTs) such as alanine aminotransferase (ALT), γ-glutamyl transferase (γ-GT), and alkaline phosphatase (ALP), and fasting plasma glucose levels, and improves insulin sensitivity [16–23]. Although pemafibrate provides uniquely favorable effects on the parameters of glucose metabolism and liver functions, few reports have addressed these issues systematically. In addition, the effects of pemafibrate on glucose metabolism may be mediated by its effects on plasma levels of fibroblast growth factor (FGF) 21, a member of the FGF family that improves energy metabolism and that is induced in response to fasting or PPARα activation [24–28].

To gain more insight into these effects of pemafibrate, we applied a post-hoc analysis to findings from six placebo-controlled studies of pemafibrate in Japanese hypertriglyceridemic subjects. We focused on how pemafibrate affected glucose metabolism and LFTs in relation to baseline values, the presence of T2D and fatty liver, and changes in TG. We also investigated the relationships between FGF21 and glucose metabolism or liver function in those patients.

## Methods

### Study design and setting

We performed a post-hoc analysis on data combined from six phase 2 and phase 3 Japanese randomized double-blind placebo-controlled studies in patients with hypertriglyceridemia [16–19, 29]. The studies enrolled a placebo group and pemafibrate groups (0.1 mg/day, 0.2 mg/day, and 0.4 mg/day). The drug was taken twice daily. The six individual studies are summarized in Table S1 in Additional file 1. Each study was approved by the Institutional Review Board for that study site. All studies were conducted in accordance with the Declaration of Helsinki after written informed consent had been obtained from each subject. This pooled analysis was approved by the Ethics Committee of Chiba University Graduate School of Medicine.

### Endpoints

The primary endpoints consisted of changes from baseline in glucose metabolism markers (fasting plasma glucose, fasting serum insulin, the homeostatic model assessment of insulin resistance [HOMA-IR], and hemoglobin A1c [HbA1c]) and the LFTs (ALP, aspartate aminotransferase [AST], ALT, γ-GT, and total bilirubin). HOMA-IR was calculated using the following formula: HOMA-IR = fasting serum insulin (µU/mL) × fasting plasma glucose (mmol/L)/22.5.

The secondary endpoints included the changes from baseline in glucose metabolism markers and LFTs as analyzed in subgroups of patients with high baseline levels for glucose metabolism or liver function, and on the presence or absence of T2D or fatty liver. The diagnosis of T2D or fatty liver was made by individual clinicians at each study site. The group with high baseline values for glucose metabolism markers consisted of patients with baseline fasting plasma glucose ≥ 7.0 mmol/L (diabetes as defined by the World Health Organization’s diagnostic criteria) or fasting serum insulin ≥ 15 µU/mL or HOMA-IR ≥ 2.5 (insulin resistance as defined by the Japanese Diabetes Treatment Guideline) [30]. The group with high baseline values for LFTs consisted of patients whose LFTs values exceeded the upper normal limits (AST > 40 U/L, ALT > 45 U/L, γ-GT > 80 U/L for males or > 30 U/L for females, respectively, ALP > 325 U/L, and total bilirubin > 20.5 µmol/L [1.2 mg/dL]). Additional secondary endpoints were the proportion of patients having high baseline LFTs that were reduced to normal levels; the percent changes in TG and HDL-C from baseline; the changes in FGF21 from baseline; the correlation between changes in TG and changes in glucose metabolism and LFTs; and the correlation between changes in FGF21 and changes in glucose metabolism and LFTs. For the safety endpoint, the incidence of adverse events and drug reactions was recorded and analyzed in each group.

### Measurements

All markers except FGF21 were measured by LSI Medience Corporation (Tokyo, Japan) using common measurement methods. FGF21 levels were measured using a human FGF21 enzyme linked immunosorbent assay (ELISA) kit (BioVendor, Brno, Czech Republic).

### Statistical analysis

The full analysis set (FAS) was used to analyze the efficacy of pemafibrate on glucose metabolism markers and LFTs. Evaluation time points were at 12 weeks last observation carried forward (LOCF). The FAS included all randomized subjects who took at least one dose of the placebo or pemafibrate and for whom a baseline and at least one post-baseline value were available to assess the efficacy endpoints. Least squares (LS) mean with a 95 % confidence interval (CI) was calculated by analysis of covariance (ANCOVA) with the baseline value as the covariate. Similar ANCOVA analyses were performed for the subgroups of the high baseline groups, the groups of patients with and without T2D, and the groups of patients with and without fatty liver. We calculated the percentage of patients whose LFT was normalized by Week 12 (LOCF), based on the above reference range at baseline, and applied Fisher’s exact test to assess the proportion in each pemafibrate group compared to the placebo group. Univariate analyses (Pearson and Spearman) were applied to the changes in TG and FGF21, and to glucose metabolism and LFTs, to obtain correlation coefficients and p-values. All patients who received at least one dose of the placebo or pemafibrate were included in the safety analysis set (SAS). Safety was analyzed descriptively. Multiplicity was not considered in any statistical analysis in this study. SAS ver. 9.4 was used for the analyses.

## Results

### Patient characteristics

The FAS population consisted of 1253 patients from the six studies, of whom 298 were randomized to placebo, 127 to pemafibrate 0.1 mg/day, 584 to pemafibrate 0.2 mg/day, and 244 to pemafibrate 0.4 mg/day (Additional file 1: Figure S1).

The mean age was 54.3 years, 14.7 % were female, 65.4 % had body mass index (BMI) ≥ 25 kg/m^2^, 35.8 % were diagnosed with T2D, and 42.6 % had fatty liver. Mean ± standard deviation (SD) values for lipids and other parameters were 3.73 ± 1.52 mmol/L in TG, HDL-C 1.13 ± 0.28 mmol/L, HbA1c 6.26 ± 0.80 %, and estimated glomerular filtration rate (eGFR) 77.9 ± 16.4 mL/min/1.73 m^2^ (Table 1).

**Table 1:** Patient characteristics at baseline

Parameter	Placebo	Pemafibrate			All
		**0.1 mg/day**	**0.2 mg/day**	**0.4 mg/day**	
n	298	127	584	244	1253
Age, years	54.9 ± 11.3	51.8 ± 11.2	55.2 ± 11.5	52.8 ± 11.3	54.3 ± 11.4
Age ≥ 65 years	62 (20.8)	16 (12.6)	130 (22.3)	40 (16.4)	248 (19.8)
Female	53 (17.8)	11 (8.7)	94 (16.1)	26 (10.7)	184 (14.7)
Body weight, kg	75.1 ± 13.4	76.7 ± 12.0	75.2 ± 13.9 ^a^	74.3 ± 12.5	75.2 ± 13.3 ^b^
BMI, kg/m^2^	26.91 ± 3.61	27.06 ± 3.61	27.08 ± 3.87 ^a^	26.25 ± 3.45	26.87 ± 3.71 ^b^
BMI ≥ 25 kg/m^2^	200 (67.1)	87 (68.5)	388 (66.4)	145 (59.4)	820 (65.4)
Type 2 diabetes	116 (38.9)	23 (18.1)	219 (37.5)	91 (37.3)	449 (35.8)
Hypertension	149 (50.0)	43 (33.9)	322 (55.1)	88 (36.1)	602 (48.0)
Fatty liver	143 (48.0)	28 (22.0)	295 (50.5)	68 (27.9)	534 (42.6)
Statin use	178 (59.7)	45 (35.4)	382 (65.4)	72 (29.5)	677 (54.0)
TG, mmol/L	3.74 ± 1.59	3.77 ± 1.21	3.72 ± 1.55	3.71 ± 1.54	3.73 ± 1.52
HDL-C, mmol/L	1.14 ± 0.25	1.10 ± 0.20	1.14 ± 0.25	1.10 ± 0.39	1.13 ± 0.28
non HDL-C, mmol/L	4.35 ± 0.82	4.52 ± 0.81	4.25 ± 0.89	4.54 ± 0.91	4.36 ± 0.88
LDL-C^c^, mmol/L	3.18 ± 0.88	3.34 ± 0.83	3.07 ± 0.90	3.37 ± 0.90	3.18 ± 0.89
TC, mmol/L	5.49 ± 0.86	5.62 ± 0.86	5.39 ± 0.91	5.65 ± 0.95	5.49 ± 0.90
HbA1c, %	6.30 ± 0.77	5.91 ± 0.66	6.31 ± 0.83	6.24 ± 0.76	6.26 ± 0.80
eGFR^d^, mL/min/1.73 m^2^	77.3 ± 17.4	76.7 ± 15.6	78.3 ± 16.4	78.6 ± 15.8	77.9 ± 16.4

Data are presented as mean ± SD for continuous parameters and n (%) for categorical parameters

*BMI* body mass index, *TG* triglyceride, *HDL-C* high-density lipoprotein cholesterol, *LDL-C* low-density lipoprotein cholesterol, *TC* total cholesterol, *HbA1c* hemoglobin A1c, *eGFR* estimated glomerular filtration rate, *SD* standard deviation, *sCr* serum creatinine

^a^ n = 583

^b^ n = 1252

^c^ LDL-C was measured using a homogeneous method (Determiner L LDL-C; Kyowa Medex, Japan)

^d^ eGFR_male_ = 194 × sCr (mg/dL)^−1.094^×age (years)^−0.287^, eGFR_female_ = 194 × sCr (mg/dL)^−1.094^×age (years)^−0.287^ × 0.739

### Glucose metabolism

**Fig. 1:**
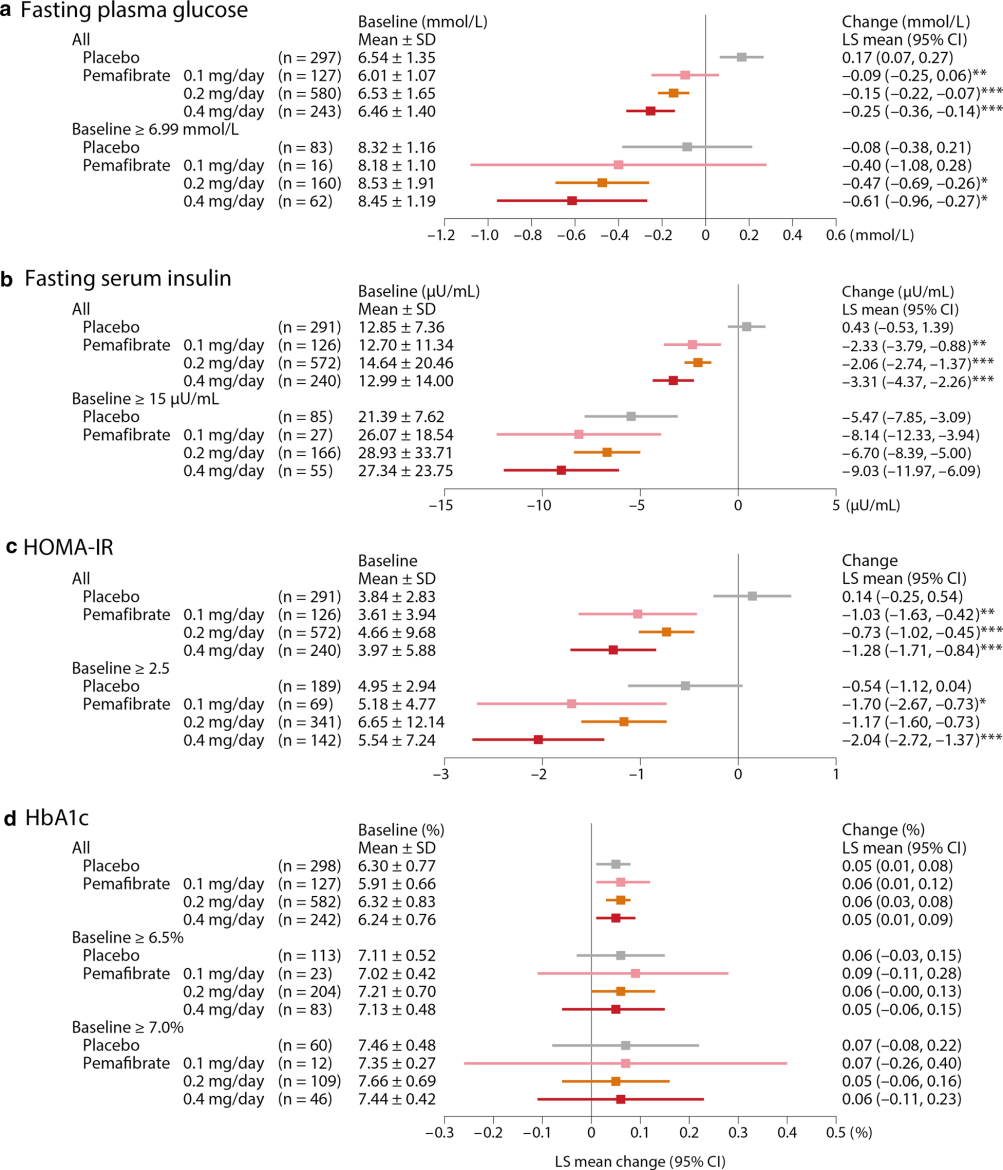
Changes in glucose metabolism markers from baseline to Week 12 (LOCF). * p < 0.05, ** p < 0.01, *** p < 0.001 vs. placebo (ANCOVA with baseline value as covariate). HOMA-IR was calculated using the following formula: HOMA-IR = fasting serum insulin (µU/mL) × fasting plasma glucose (mmol/L)/22.5. *HOMA-IR* homeostatic model assessment of insulin resistance, *HbA1c* hemoglobin A1c, *SD* standard deviation, *LS* least squares, *CI* confidence interval, *LOCF* last observation carried forward, *ANCOVA* analysis of covariance

No significant changes were noted in fasting plasma glucose, fasting serum insulin, or HOMA-IR in the placebo group. However, in all populations, these three measurements decreased significantly for pemafibrate compared to placebo. The greatest decrease was seen at the dose of 0.4 mg/day; in that group, fasting plasma glucose values for LS mean (95 % CI) differed from baseline by − 0.25 (− 0.36–−0.14) mmol/L (p < 0.001 vs. placebo); fasting serum insulin by − 3.31 (− 4.37–−2.26) µU/mL (p < 0.001); and HOMA-IR by − 1.28 (− 1.71–−0.84) (p < 0.001) (Fig. 1).

The patients with higher baseline values showed greater decreases from baseline in fasting plasma glucose, fasting serum insulin, and HOMA-IR in all pemafibrate groups. Those differences were greatest in the pemafibrate 0.4 mg/day group: fasting plasma glucose values for LS mean (95 % CI) differed from baseline by − 0.61 (− 0.96–−0.27) mmol/L (p < 0.05 vs. placebo); fasting serum insulin by − 9.03 (− 11.97–−6.09) µU/mL (p = 0.065); and HOMA-IR by − 2.04 (− 2.72–−1.37) (p < 0.001) (Fig. 1).

For patients without T2D, decreases in fasting plasma glucose, fasting serum insulin, and HOMA-IR were significantly greater than placebo at all pemafibrate doses. For patients with T2D, decreases in fasting plasma glucose were significantly greater than placebo in groups treated with 0.2 mg/day or above, and decreases from baseline in fasting serum insulin and HOMA-IR were significantly greater in the 0.4 mg/day group. Regardless of the T2D status, these glucose metabolism markers showed the greatest decrease in the 0.4 mg/day group (Additional file 1: Figure S2).

In the absence of fatty liver, decreases in fasting plasma glucose, fasting serum insulin, and HOMA-IR were significantly greater than placebo at all pemafibrate doses. For patients with fatty liver, decreases in fasting plasma glucose and fasting serum insulin were significantly greater than placebo in groups treated with 0.2 mg/day or above, and decreases in HOMA-IR were significantly greater in the 0.4 mg/day group. Regardless of fatty liver status, these glucose metabolism markers showed the greatest decrease in the 0.4 mg/day group (Additional file 1: Figure S3).

For HbA1c, our analysis showed no statistically significant difference between pemafibrate and placebo, both overall and when stratified by HbA1c at baseline (Fig. 1) or by patients with and without T2D (Additional file 1: Figure S2) or with and without fatty liver (Additional file 1: Figure S3).

### LFTs

**Fig. 2:**
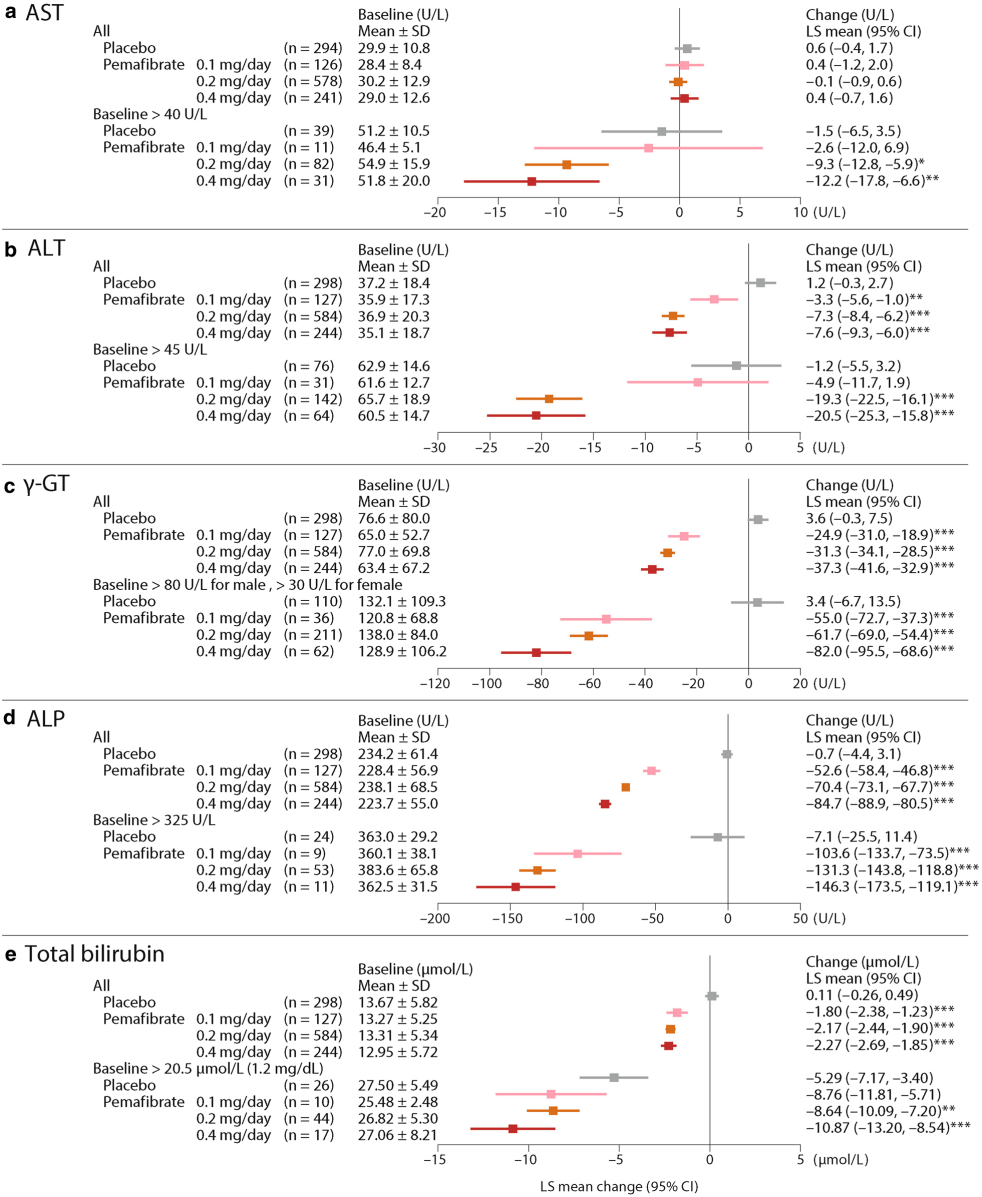
Changes in liver function tests from baseline to Week 12 (LOCF). * p < 0.05, ** p < 0.01, *** p < 0.001 vs. placebo (ANCOVA with baseline value as covariate). *AST* aspartate aminotransferase, *ALT* alanine aminotransferase, *γ-GT* γ-glutamyl transferase, *ALP* alkaline phosphatase, *SD* standard deviation, *LS* least squares, *CI* confidence interval, *LOCF* last observation carried forward, *ANCOVA* analysis of covariance

No significant decreases in AST, ALT, γ-GT, ALP, or total bilirubin were noted among any patients in the placebo group. However, in all populations, the LFTs except for AST decreased significantly for all pemafibrate groups compared to placebo. The greatest decrease from baseline was seen in the pemafibrate 0.4 mg/day group: for ALT, LS mean (95 % CI) values differed by − 7.6 (− 9.3–−6.0) U/L (p < 0.001 vs. placebo), γ-GT by − 37.3 (− 41.6–−32.9) U/L (p < 0.001), ALP by − 84.7 (− 88.9–−80.5) U/L (p < 0.001), and total bilirubin by − 2.27 (− 2.69–−1.85) µmol/L (p < 0.001). Results for AST showed no difference from placebo in any of the pemafibrate groups (Fig. 2).

Greater decreases in AST, ALT, γ-GT, ALP, and total bilirubin were noted in those patients with high baseline values (Fig. 2). The greatest decrease from baseline was seen at pemafibrate 0.4 mg/day; for ALT, LS mean (95 % CI) values differed by − 20.5 (− 25.3–−15.8) U/L (p < 0.001 vs. placebo), γ-GT by − 82.0 (− 95.5–−68.6) U/L (p < 0.001), ALP by − 146.3 (− 173.5–−119.1) U/L (p < 0.001), and total bilirubin by − 10.87 (− 13.20–−8.54) µmol/L (p < 0.001). In that group, AST values differed by − 12.2 (− 17.8–−6.6) U/L (p < 0.01) (Fig. 2).

**Fig. 3:**
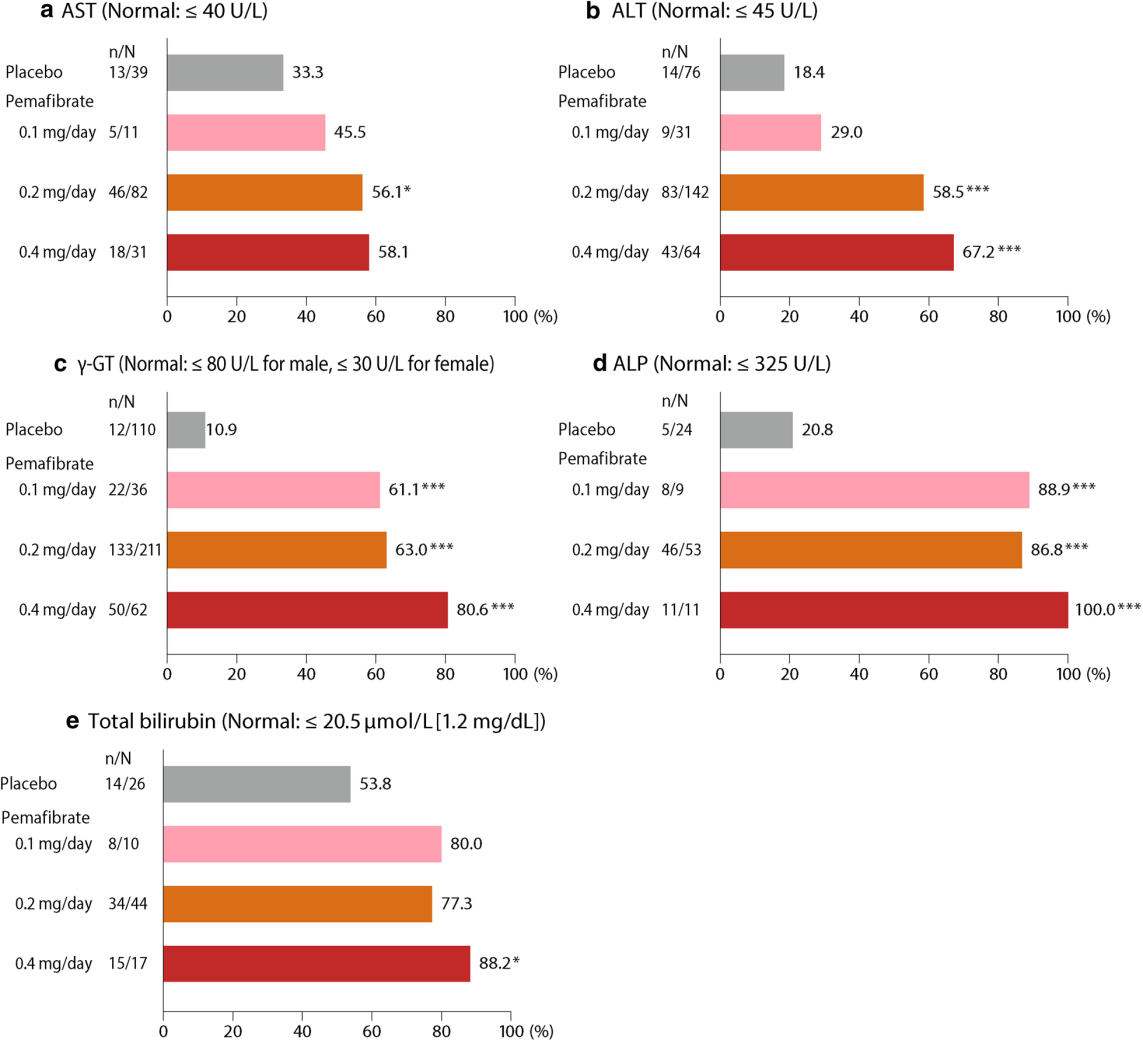
Percentage of patients whose liver function tests were normalized at Week 12 (LOCF) from above reference range at baseline. * p < 0.05, *** p < 0.001 vs. placebo (Fisher’s exact test). *AST* aspartate aminotransferase, *ALT* alanine aminotransferase, *γ-GT* γ-glutamyl transferase, *ALP* alkaline phosphatase, *LOCF* last observation carried forward

In the pemafibrate 0.2 mg/day and 0.4 mg/day groups, a significantly higher proportion of the patients whose baseline ALT, γ-GT, and ALP levels exceeded the upper normal limits had achieved normal levels at Week 12 (LOCF) than in the placebo group. The greatest proportion of improved patients was in the 0.4 mg/day group: AST (58.1 %), ALT (67.2 %), γ-GT (80.6 %), ALP (100 %), and total bilirubin (88.2 %) (Fig. 3).

For patients without T2D, decreases in ALT, γ-GT, ALP, and total bilirubin were significantly greater than placebo at all pemafibrate doses. For patients with T2D, decreases in γ-GT, ALP, and total bilirubin were significantly greater than placebo in all pemafibrate groups, and decreases in ALT were significantly greater in groups treated with 0.2 mg/day or above. With the exception of ALT in patients without T2D, these decreases were greatest in the 0.4 mg/day group (Additional file 1: Figure S4).

In the absence of fatty liver, the decreases in ALT, γ-GT, ALP, and total bilirubin were significantly greater than placebo at all pemafibrate doses. For patients with fatty liver, the decreases in γ-GT, ALP, and total bilirubin were significantly greater than placebo in all pemafibrate groups, and the decreases in ALT were significantly greater in groups treated with 0.2 mg/day or more. With the exception of ALT in patients without fatty liver, these LFTs showed the greatest decrease in the 0.4 mg/day group (Additional file 1: Figure S5).

### TG and HDL-C

**Fig. 4:**
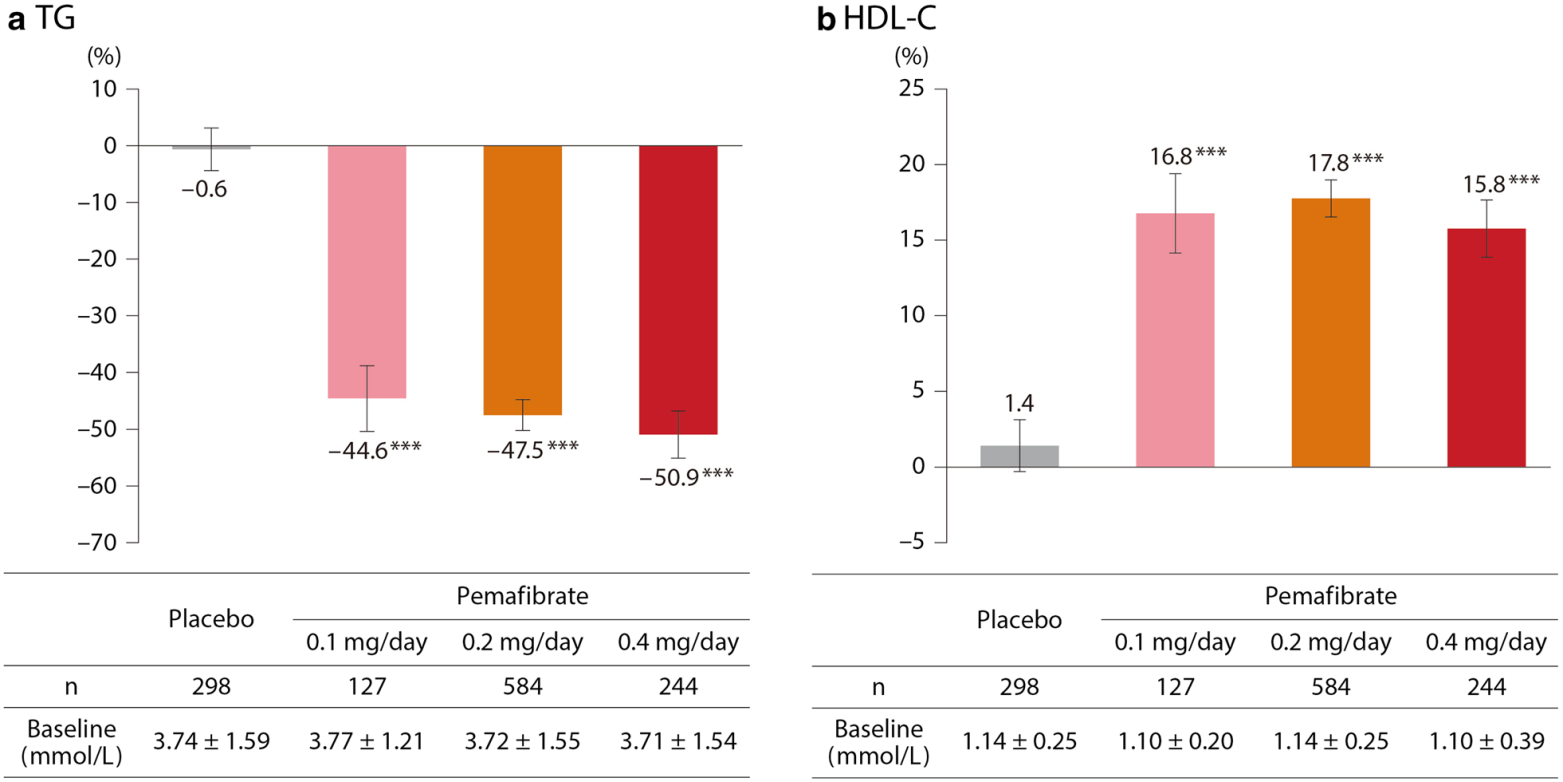
Percentage changes in TG and HDL-C levels. Baseline levels indicate mean ± SD, and bars indicate LS mean percentage change (95 % CI) from baseline to Week 12 (LOCF). *** p < 0.001 vs. placebo (ANCOVA with baseline value as covariate). *TG* triglyceride, *HDL-C* high-density lipoprotein cholesterol, *SD* standard deviation, *LS* least squares, *CI* confidence interval, *LOCF* last observation carried forward, *ANCOVA* analysis of covariance

TG levels decreased significantly for all pemafibrate doses compared with placebo (LS mean values for percentage changes from baseline: −44.6 % for 0.1 mg/day, − 47.5 % for 0.2 mg/day, − 50.9 % for 0.4 mg/day, p < 0.001 for each vs. placebo) (Fig. 4). HDL-C levels increased significantly for all pemafibrate doses compared to baseline (LS mean values for percentage changes: 16.8 % for 0.1 mg/day, 17.8 % for 0.2 mg/day, 15.8 % for 0.4 mg/day, p < 0.001 for each vs. placebo) (Fig. 4).

### FGF21

**Fig. 5:**
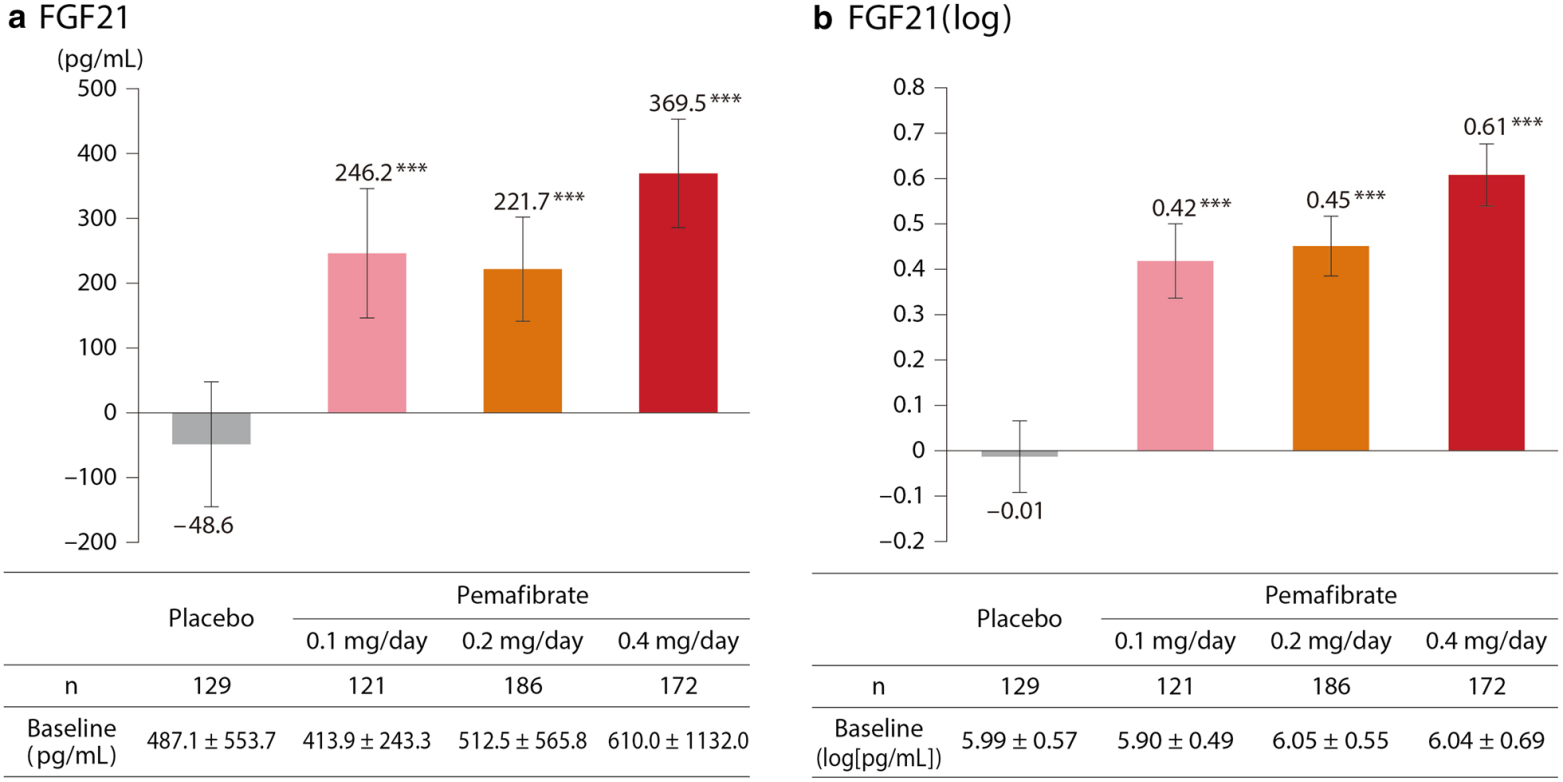
Changes in FGF21 levels. Baseline levels indicate mean ± SD, and bars indicate LS mean change (95 % CI) from baseline to Week 12 (LOCF). *** p < 0.001 vs. placebo (ANCOVA with baseline value as covariate). *FGF21* fibroblast growth factor 21, *SD* standard deviation, *LS* least squares, *CI* confidence interval, *LOCF* last observation carried forward, *ANCOVA* analysis of covariance

FGF21 increased significantly for all pemafibrate doses compared to placebo. The greatest increase was noted at pemafibrate 0.4 mg/day, which changed from baseline by + 369.5 pg/mL, p < 0.001 vs. placebo (Fig. 5).

### Coefficient of correlation between changes in TG or FGF21 and the glucose metabolism markers or LFTs

There were no substantial correlations between changes in either TG or FGF21 and any of the glucose metabolism markers (fasting plasma glucose, fasting serum insulin, HOMA-IR) (Additional file 1: Table S2).

We found significant positive correlations, but with relatively small coefficients of correlation, for changes in γ-GT and TG (Pearson’s product moment 0.164, Spearman’s rank 0.178) and in ALP and TG (Pearson’s product moment 0.156, Spearman’s rank 0.155). There was a significant negative correlation for changes in total bilirubin and FGF21, again with a relatively small coefficient of correlation (Pearson’s product moment − 0.169, Spearman’s rank − 0.204) (Additional file 1: Table S3). In comparison to all patients, patients with high baseline values showed greater positive correlations for changes in γ-GT and TG (Pearson’s product moment 0.240, Spearman’s rank 0.222). For changes in ALP and FGF21, the correlation coefficient was higher in the high baseline patients than in all patients, but did not reach significance (Pearson’s product moment − 0.244, Spearman’s rank − 0.229). Similarly, for changes in total bilirubin and FGF21, the correlation coefficient was higher in the high baseline patients than in all patients but did not reach significance (Pearson’s product moment − 0.313, Spearman’s rank − 0.239) (Additional file 1: Table S3).

### Adverse events and adverse drug reactions

Adverse events occurred in 43.0 % (128/298) of patients in the placebo group, 44.1 % (56/127) in the pemafibrate 0.1 mg/day group, 41.4 % (242/584) in the 0.2 mg/day group, and 38.2 % (94/246) in the 0.4 mg/day group. Serious adverse events (SAEs) developed in two patients (0.7 %, embolic stroke and angina pectoris) in the placebo group, three patients (2.4 %, acute myocardial infarction, cervical cancer, and upper limb fracture) in the 0.1 mg/day group, 10 patients (1.7 %; one patient each with ureteral calculus, bile duct stone, malignant lung neoplasm, colon cancer, hematoma of the abdominal wall, lumbar spinal stenosis, varicose vein surgery, and diabetes mellitus, and two patients with enterocolitis) in the 0.2 mg/day group, and two patients (0.8 %, ureteral calculus and myocardial infarction) in the 0.4 mg/day group, plus one death (pulmonary embolism) in the 0.4 mg/day group [15].

## Discussion

Our study revealed that glucose metabolism markers (fasting plasma glucose, fasting serum insulin, HOMA-IR) and LFTs (ALT, γ-GT, ALP, and total bilirubin) were significantly reduced by pemafibrate. The change was particularly notable in the subgroups with high baseline values. In the high baseline LFTs subgroup, the 0.4 mg/day group had the highest proportion of patients who achieved normal LFT values. These findings were consistent with previous studies, which also showed pemafibrate-induced improvements in TG and HDL-C and increases in FGF21. Of note, diabetes and NAFLD have been found to correlate closely with abnormal lipid metabolism [1, 2], and pemafibrate not only improves lipid metabolism, but also may beneficially affect glucose metabolism and liver function at a high dose (0.4 mg/day). Related to those findings, we previously published the results of pooled analysis showing that high-dose pemafibrate was associated with marked reductions in ApoB48, ApoC3, ApoC3/ApoC2, small low-density lipoprotein cholesterol (LDL-C), and fibrinogen [15].

In a previous study using diet-induced obese mice, oral glucose tolerance tests (OGTT) showed that post-challenge serum insulin was reduced by pemafibrate, suggesting that pemafibrate improves insulin sensitivity [31]. Findings from another study indicated that pemafibrate enhanced the expression of the ATP-binding cassette transporter A1 (ABCA1) and reduced the level of 8-hydroxy-2’-deoxyguanosine (8-OHdG) in pancreatic β cells, suggesting that pemafibrate may improve insulin secretory capacity in diabetic patients by mitigating lipotoxicity and reducing oxidative stress in the pancreas [32, 33]. Pemafibrate use has also been associated with improved hepatic glucose uptake capacity in patients with hypertriglyceridemia, as evaluated by the glucose clamp technique [29]. Although drugs that activate PPARα do not have a consistent effect on glucose metabolism [34–36], we noted improvement in both fasting plasma glucose and insulin sensitivity in this study. There was no notable correlation between changes in glucose metabolism and changes in TG in our study, suggesting that pemafibrate may possibly improve glucose metabolism independently of reduction in TG. Previous studies of pemafibrate in T2D have provided somewhat inconsistent behaviors in HbA1c, glycoalbumin, plasma glucose, and serum insulin [19, 22, 37]. Similarly, our pooled analysis indicated that the increase in HbA1c tended to be about the same for pemafibrate and placebo, even in groups that showed pemafibrate-induced reductions in plasma glucose and serum insulin compared to the placebo control. However, when we consider that HbA1c indicates the plasma glucose over the previous one to two months, and that the data analyzed from our study was collected during a relatively short period of 12 weeks, these apparent inconsistencies may simply represent a divergence between HbA1c and current changes in plasma glucose and serum insulin. It is possible that worsening of postprandial glucose might mitigate the improvement in fasting glucose, neutralizing the effect on HbA1c; however, our previous study of pemafibrate did not show worsening of postprandial glucose level [19]. It is also possible that the natural rise in HbA1c mitigates the expected improvement in HbA1c based on blood glucose values.

Whether improved LFTs are related to improvement in non-alcoholic steatohepatitis (NASH) and NAFLD remains unproven at this point. However, obeticholic acid, a farnesoid X nuclear receptor (FXR) ligand that modulates lipid metabolism, has been associated with improvement in the liver histology of patients with NASH, and also with significant decreases in AST, ALT, γ-GT, and total bilirubin [38]. The effects of pemafibrate on reduction of liver fat and suppression of liver fibrogenesis have been studied in rodent NASH models [39]. A phase 2 study of pemafibrate, currently in process, is using noninvasive methods to measure liver fat content and liver stiffness (ClinicalTrials.gov Identifier: NCT03350165). This study, which focuses on the 0.4 mg/day pemafibrate dose, enrolled patients with liver fat content of 10 % or above, liver stiffness of 2.5 kPa or above, and high ALT. The study is expected to show meaningful effects on liver fat, liver function values, and liver fibrosis.

In addition, pemafibrate may be useful in the treatment of primary biliary cholangitis because, like obeticholic acid, fenofibrate and bezafibrate [40–42], pemafibrate decreases both ALP and total bilirubin. The expression of genes related to bile acid metabolism is included in the factors regulated by PPARα, and PPARα agonists may improve cholestasis [43]. Pemafibrate also provides more potent ALP reduction than the conventional PPARα agonist fenofibrate [16, 20], and is expected to be a useful treatment option for primary biliary cholangitis. In our study, the subgroups with high baseline values showed weak positive correlations between changes in TG and changes in ALP and γ-GT, suggesting that decreased TG may contribute to decreases in γ-GT and ALP. However, this study failed to clearly demonstrate a possible role for TG reduction in improving LFTs. Data from pemafibrate case reports of patients with severe hypertriglyceridemia (TG exceeding 1000 mg/dL) showed liver function test values within the normal range [44], suggesting that TG does not necessarily correlate with liver function values.

FGF21 plays a critical role in metabolic regulation [45, 46], and its analog has reportedly improved glucose and lipid metabolism and NAFLD in clinical trials [47, 48]. In our study, the pemafibrate groups showed increased FGF21, with the greatest increase in the 0.4 mg/day group. That group also experienced the greatest reduction in TG and the greatest improvement in LFTs. After administration of an FGF21 analog, FGF21 blood levels were reported to be within a range of 17.5 to 150 ng/mL [47]. However, in our study, FGF21 blood levels ranged from 610.0 pg/mL to approximately 1000 pg/mL (= 1 ng/mL), even for pemafibrate 0.4 mg/day (changed by + 369.5 pg/mL). These results clearly did not reach the level achieved by the FGF21 analog. In addition, although patients with high baseline values tended to show a negative correlation between changes in blood FGF21 and changes in ALP/total bilirubin, the relationship was not statistically significant. However, it remains possible that pemafibrate-induced increases in FGF21 may have beneficial effects on the liver, because FGF21 may affect the liver or local nerve tissue in a paracrine manner [46]. Pemafibrate is also expected to ameliorate NASH by improving lipid turnover, promoting energy metabolism, and reducing insulin resistance and inflammation [39]. Future research is needed to understand how pemafibrate and FGF21 are related to lipid metabolism, glucose metabolism, and liver function improvement.

## Limitations

This study had some limitations. It was a post-hoc analysis of pooled data from multiple prospective randomized controlled trials. The study was limited to Japanese hypertriglyceridemic subjects, so the possibility of racial differences needs to be addressed. Further pharmacophysiological research is needed to clarify how pemafibrate improved glucose metabolism and LFTs. In addition, our study did not resolve the question of whether improvement in LFT values would lead to improvement of NASH or NAFLD; we hope that a phase 2 study of pemafibrate on NAFLD, which is currently underway, will provide answers to these questions.

## Conclusions

In addition to improving TG and HDL-C, pemafibrate favorably affected glucose metabolism markers, LFTs, and FGF21. These effects were particularly notable among patients with high baseline values for glucose and liver function, and tended to be greatest in the 0.4 mg/day group. Mechanisms of action should be further explored for these effects of pemafibrate.

## Supplementary Information

**Additional file 1: Table S1.** Summary of six randomized double-blind placebo-controlled clinical trials of pemafibrate. **Table S2.** Correlations between changes in fasting plasma glucose, fasting serum insulin and HOMA-IR, and changes in TG and FGF21 from baseline to Week 12 (LOCF): correlation coefficients from Pearson and Spearman analyses, with the respective p-values. **Table S3.** Correlations between changes in liver function tests and changes in TG and FGF21 from baseline to Week 12 (LOCF): correlation coefficients from Pearson and Spearman analyses, with the respective p-values. **Figure S1.** Subject disposition. **Figure S2.** Changes in glucose metabolism markers, from baseline to Week 12 (LOCF), by presence or absence of type 2 diabetes. **Figure S3.** Changes in glucose metabolism markers, from baseline to Week 12 (LOCF), by presence or absence of fatty liver. **Figure S4.** Changes in liver function tests, from baseline to Week 12 (LOCF), by presence or absence of type 2 diabetes. **Figure S5.** Changes in liver function tests, from baseline to Week 12 (LOCF), by presence or absence of fatty liver.

## Data Availability

Data in this study were used under license, and are not publicly available.

## Abbreviations

T2D: Type 2 diabetes
NAFLD: Non-alcoholic fatty liver disease
PPAR: Peroxisome proliferator-activated receptor
TG: Triglyceride
SPPARMα: Selective PPAR α modulator
HDL-C: High-density lipoprotein cholesterol
LFT: Liver function test
ALT: Alanine aminotransferase
γ-GT: γ-Glutamyl transferase
ALP: Alkaline phosphatase
FGF: Fibroblast growth factor
HOMA-IR: Homeostatic model assessment of insulin resistance
HbA1c: Hemoglobin A1c
AST: Aspartate aminotransferase
ELISA: Enzyme linked immunosorbent assay
FAS: Full analysis set
LOCF: Last observation carried forward
LS: Least squares
CI: Confidence interval
ANCOVA: Analysis of covariance
SAS: Safety analysis set
BMI: Body mass index
SD: Standard deviation
eGFR: Estimated glomerular filtration rate
SAE: Serious adverse event
LDL-C: Low-density lipoprotein cholesterol
OGTT: Oral glucose tolerance test
ABCA1: ATP-binding cassette transporter A1
8-OHdG: 8-Hydroxy-2’-deoxyguanosine
NASH: Non-alcoholic steatohepatitis
FXR: Farnesoid X nuclear receptor
