# Supplementary material for: Effects of pemafibrate on glucose metabolism markers and liver function tests in patients with hypertriglyceridemia: a pooled analysis of six phase 2 and phase 3 randomized double‐blind placebo‐controlled clinical trials
Source: Cardiovasc Diabetol. 2021 May 4;20:96. doi: 10.1186/s12933-021-01291-w (PMC8097867; doi:10.1186/s12933-021-01291-w)
Supplement: Supplementary file 1 — Additional file 1: Table S1. Summary of six randomized double-blind placebo-controlled clinical trials of pemafibrate. Table S2. Correlations between changes in fasting plasma glucose, fasting serum insulin and HOMA-IR, and changes in TG and FGF21 from baseline to Week 12 (LOCF): correlation coefficients from Pearson and Spearman analyses, with the respective p-values. Table S3. Correlations between changes in liver function tests and changes in TG and FGF21 from baseline to Week 12 (LOCF): correlation coefficients from Pearson and Spearman analyses, with the respective p-values. Figure S1. Subject disposition. Figure S2. Changes in glucose metabolism markers, from baseline to Week 12 (LOCF), by presence or absence of type 2 diabetes. Figure S3. Changes in glucose metabolism markers, from baseline to Week 12 (LOCF), by presence or absence of fatty liver. Figure S4. Changes in liver function tests, from baseline to Week 12 (LOCF), by presence or absence of type 2 diabetes. Figure S5. Changes in liver function tests, from baseline to Week 12 (LOCF), by presence or absence of fatty liver. [file 12933_2021_1291_MOESM1_ESM.pdf]

Additional file 1

Effects of pemafibrate on glucose metabolism markers and liver function tests in patients with hypertriglyceridemia: A pooled analysis of six phase 2 and phase 3 randomized double-blind placebo-controlled clinical trials

Supplementary Tables

Table S1 Summary of six randomized double-blind placebo-controlled clinical trials of pemafibrate ----------------- 2

Table S2 Correlations between changes in fasting plasma glucose, fasting serum insulin and HOMA-IR, and changes in TG and FGF21 from baseline to Week 12 (LOCF): correlation coefficients from Pearson and Spearman analyses, with the respective p-values -- 3

Table S3 Correlations between changes in liver function tests and changes in TG and FGF21 from baseline to Week 12 (LOCF): correlation coefficients from Pearson and Spearman analyses, with the respective p-values -------------- 4

Supplementary Figures

Figure S1 Subject disposition -- 5

Figure S2 Changes in glucose metabolism markers, from baseline to Week 12 (LOCF), by presence or absence of type 2 diabetes -- 6

Figure S3 Changes in glucose metabolism markers, from baseline to Week 12 (LOCF), by presence or absence of fatty liver -- 7

Figure S4 Changes in liver function tests, from baseline to Week 12 (LOCF), by presence or absence of type 2 diabetes --- 8

Figure S5 Changes in liver function tests, from baseline to Week 12 (LOCF), by presence or absence of fatty liver -- 9

Table S1 Summary of six randomized double-blind placebo-controlled clinical trials of pemafibrate [Yamashita S, et al. Int J Mol Sci. 2019;20(22):5537.]

Study No.	Dose	Inclusion criteria regarding lipid parameters	Primary outcomes	n [a]	Duration	Reference
K-877-04<br>Dose finding	Placebo<br>Pemafibrate 0.05, 0.1, 0.2, or 0.4 mg/day<br>Fenofibrate 100 mg/day	TG: 2.26-5.65 mmol/L (200-500 mg/dL)<br>HDL-C:<br>< 1.29 mmol/L (50 mg/dL) (Male),<br>< 1.42 mmol/L (55 mg/dL) (Female)	Efficacy: Percent change in fasting TG<br>Safety: Incidence of AEs and ADRs	224	12 weeks	Ishibashi S, et al. Atherosclerosis. 2016;249:36-43.
K-877-09<br>Compared to fenofibrate	Placebo<br>Pemafibrate 0.1, 0.2, or 0.4 mg/day<br>Fenofibrate 100 or 200 mg/day	TG: 2.26-11.29 mmol/L (200-1000 mg/dL)<br>HDL-C:<br>< 1.29 mmol/L (50 mg/dL) (Male),<br>< 1.42 mmol/L (55 mg/dL) (Female)	Efficacy: Percent change in fasting TG<br>Safety: Incidence of AEs and ADRs	526	12 weeks	Arai H, et al. J Atheroscler Thromb. 2018;25:521-38.
K-877-13<br>Add-on to pitavastatin	Placebo<br>Pemafibrate 0.1, 0.2, or 0.4 mg/day	TG: 2.26-11.29 mmol/L (200-1000 mg/dL)<br>Non HDL-C: ≥ 3.88 mmol/L (150 mg/dL)	Efficacy: Percent change in fasting TG<br>Safety: Incidence of AEs and ADRs	188	12 weeks	Arai H, et al. Atherosclerosis. 2017;261:144-52.
K-877-15<br>Add-on to any statin	Placebo<br>Pemafibrate 0.2 or 0.2 (0.4) [b] mg/day	TG: 2.26-11.29 mmol/L (200-1000 mg/dL)	Efficacy: Percent change in fasting TG<br>Safety: Incidence of AEs and ADRs	423	24 weeks	Arai H, et al. Atherosclerosis. 2017;261:144-52.
K-877-16<br>Type 2 diabetes	Placebo [c]<br>Pemafibrate 0.2 or 0.4 mg/day	TG: 1.69-11.29 mmol/L (150-1000 mg/dL)	Efficacy: Percent change in fasting TG<br>Safety: Incidence of AEs and ADRs	166	24-52 weeks	Araki E, et al. Diabetes Care. 2018;41:538-46.
K-877-19<br>Glucose clamp	Placebo<br>Pemafibrate 0.4 mg/day	TG: 2.26-5.65 mmol/L (200-500 mg/dL)	Efficacy: Change in splanchnic glucose uptake<br>Safety: Incidence of AEs and ADRs	27	12 weeks	Matsuba I, et al. J Diabetes Investig. 2018;9:1323-32.

[a] the number of randomized patients, [b] up-titrating from pemafibrate 0.2 mg/day to 0.4 mg/day after Week 12 if TG levels ≥ 1.69 mmol/L (150 mg/dL) at Week 8, [c] switching from placebo to pemafibrate 0.2 mg/day after Week 24

TG triglyceride, *HDL-C* high-density lipoprotein cholesterol, *AEs* adverse events, *ADRs* adverse drug reactions

Table S2 Correlations between changes in fasting plasma glucose, fasting serum insulin and HOMA-IR, and changes in TG and FGF21 from baseline to Week 12 (LOCF): correlation coefficients from Pearson and Spearman analyses, with the respective p-values

		**Changes in TG**				**Changes in FGF21**			
**Changes**		**Pearson's product moment correlation coefficient**	**p**	**Spearman's rank correlation coefficient**	**p**	**Pearson's product moment correlation coefficient**	**p**	**Spearman's rank correlation coefficient**	**p**
Fasting plasma glucose	All	–0.044	0.180	–0.008	0.809	0.023	0.609	0.019	0.682
	Baseline ≥ 6.99 mmol/L	–0.094	0.148	0.041	0.533	0.134	0.252	0.253	0.029
Fasting serum insulin	All	–0.005	0.885	–0.013	0.700	0.027	0.558	0.013	0.780
	Baseline ≥ 15 μU/mL	0.034	0.597	0.002	0.973	0.139	0.131	0.127	0.168
HOMA-IR	All	–0.008	0.796	–0.014	0.678	0.034	0.466	0.007	0.875
	Baseline ≥ 2.5	–0.002	0.967	–0.033	0.435	0.062	0.314	0.060	0.330

Patients in the pemafibrate 0.1 mg/day, 0.2 mg/day, and 0.4 mg/day groups

HOMA-IR was calculated using the following formula: HOMA-IR = fasting serum insulin (μU/mL) × fasting plasma glucose (mmol/L)/22.5

TG triglyceride, *FGF21* fibroblast growth factor 21, *LOCF* last observation carried forward, *HOMA-IR* homeostatic model assessment of insulin resistance

Table S3 Correlations between changes in liver function tests and changes in TG and FGF21 from baseline to Week 12 (LOCF): correlation coefficients from Pearson and Spearman analyses, with the respective p-values

		Changes in TG				Changes in FGF21			
**Changes**		**Pearson's product moment correlation coefficient**	**p**	**Spearman's rank correlation coefficient**	**p**	**Pearson's product moment correlation coefficient**	**p**	**Spearman's rank correlation coefficient**	**p**
AST	All	0.081	0.013	0.059	0.070	0.019	0.680	0.157	< 0.001
	Baseline > 40 U/L	0.091	0.316	0.181	0.044	–0.146	0.340	0.085	0.577
ALT	All	0.083	0.010	0.066	0.041	0.021	0.653	0.119	0.009
	Baseline > 45 U/L	0.106	0.105	0.107	0.102	–0.026	0.784	0.105	0.261
γ-GT	All	0.164	< 0.001	0.178	< 0.001	–0.142	0.002	0.149	0.001
	Baseline > 80 U/L for male, > 30 U/L for female	0.240	< 0.001	0.222	< 0.001	–0.298	< 0.001	0.125	0.161
ALP	All	0.156	< 0.001	0.155	< 0.001	–0.050	0.276	–0.063	0.169
	Baseline > 325 U/L	0.156	0.187	0.192	0.104	–0.244	0.186	–0.229	0.215
Total bilirubin	All	–0.059	0.068	–0.012	0.701	–0.169	< 0.001	–0.204	< 0.001
	Baseline > 20.5 μmol/L (1.2 mg/dL)	0.004	0.971	–0.083	0.491	–0.313	0.072	–0.239	0.173

Patients in the pemafibrate 0.1 mg/day, 0.2 mg/day, and 0.4 mg/day group

TG triglyceride, *FGF* fibroblast growth factor, *LOCF* last observation carried forward, *AST* aspartate aminotransferase, *ALT* alanine aminotransferase, *γ-GT* γ-glutamyl transferase, *ALP* alkaline phosphatase

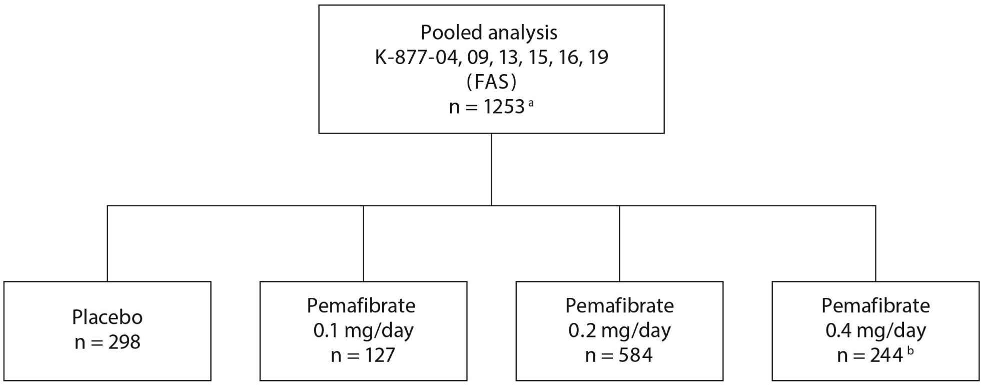

Figure S1 Subject disposition. [a] Safety analysis set: n = 1255, [b] Safety analysis set: n = 246. *FAS* full analysis set

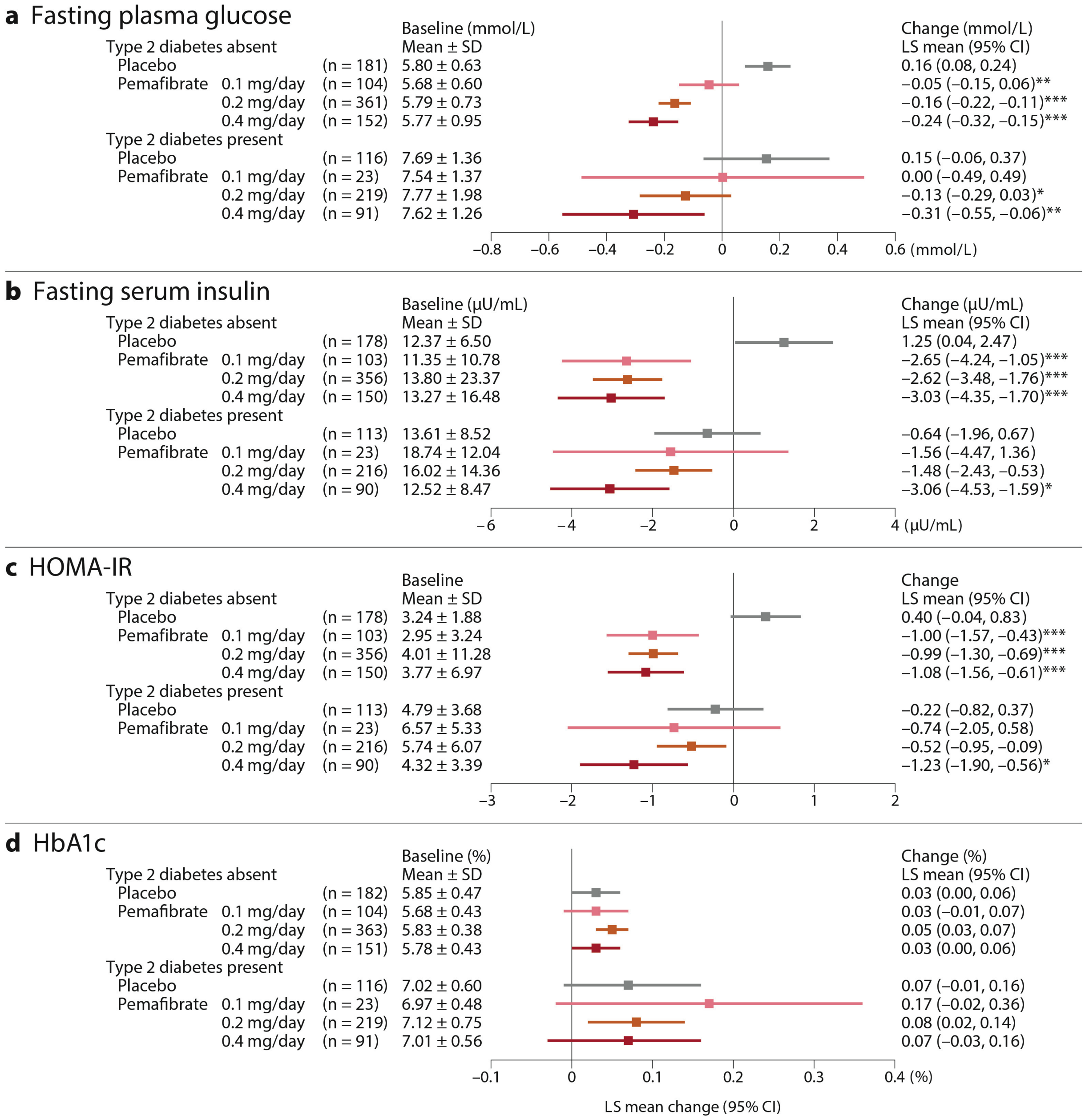

Figure S2 Changes in glucose metabolism markers, from baseline to Week 12 (LOCF), by presence or absence of type 2 diabetes. * $p < 0.05$, ** $p < 0.01$, *** $p < 0.001$ vs. placebo (ANCOVA with baseline value as covariate). HOMA-IR was calculated using the following formula: HOMA-IR = fasting serum insulin (μU/mL) × fasting plasma glucose (mmol/L)/22.5. *HOMA-IR* homeostatic model assessment of insulin resistance, *HbA1c* hemoglobin A1c, *SD* standard deviation, *LS* least squares, *CI* confidence interval, *LOCF* last observation carried forward, *ANCOVA* analysis of covariance

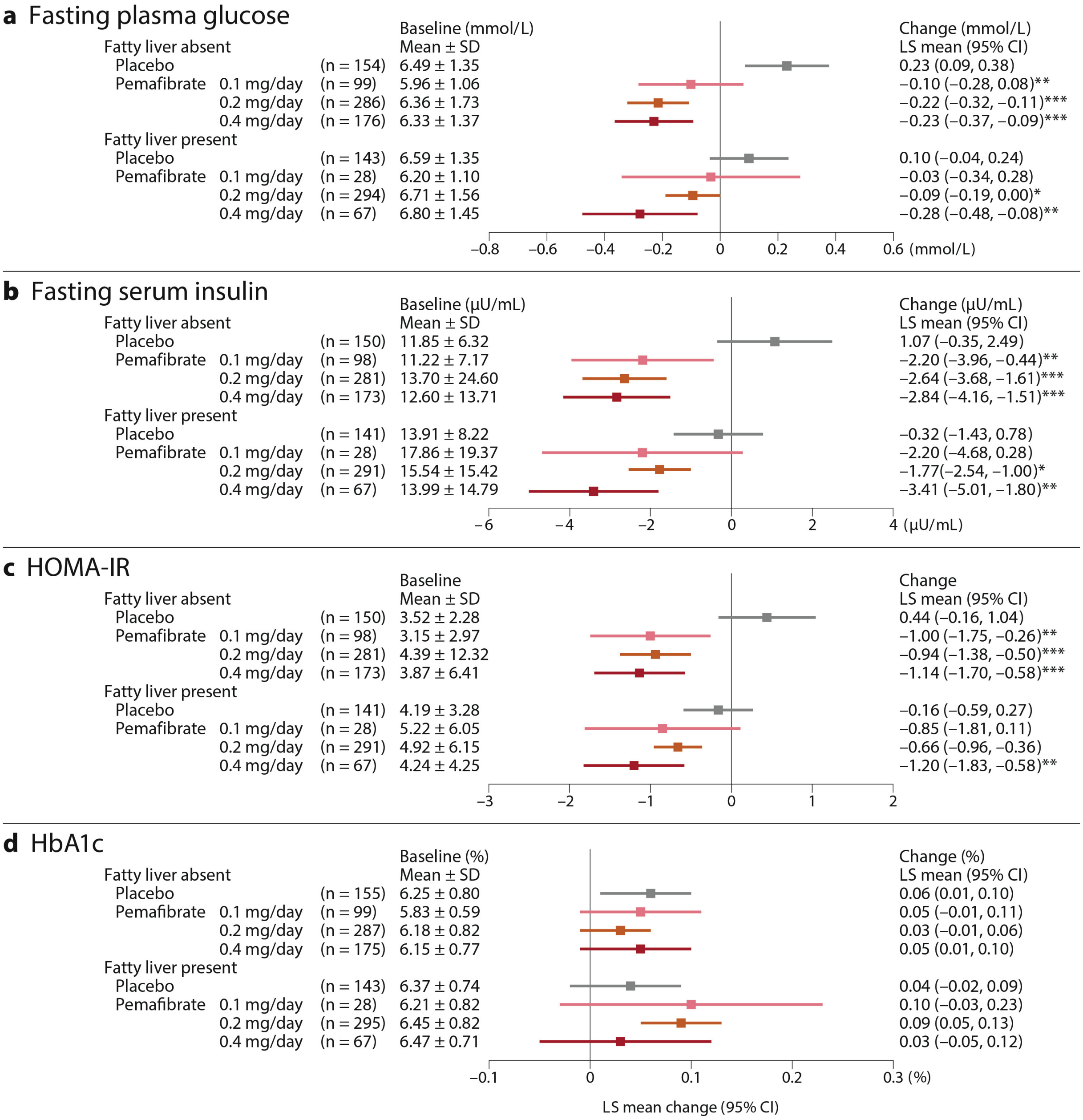

Figure S3 Changes in glucose metabolism markers, from baseline to Week 12 (LOCF), by presence or absence of fatty liver. * $p < 0.05$, ** $p < 0.01$, *** $p < 0.001$ vs. placebo (ANCOVA with baseline value as covariate). HOMA-IR was calculated using the following formula: HOMA-IR = fasting serum insulin (μU/mL) × fasting plasma glucose (mmol/L)/22.5. *HOMA-IR* homeostatic model assessment of insulin resistance, *HbA1c* hemoglobin A1c, *SD* standard deviation, *LS* least squares, *CI* confidence interval, *LOCF* last observation carried forward, *ANCOVA* analysis of covariance

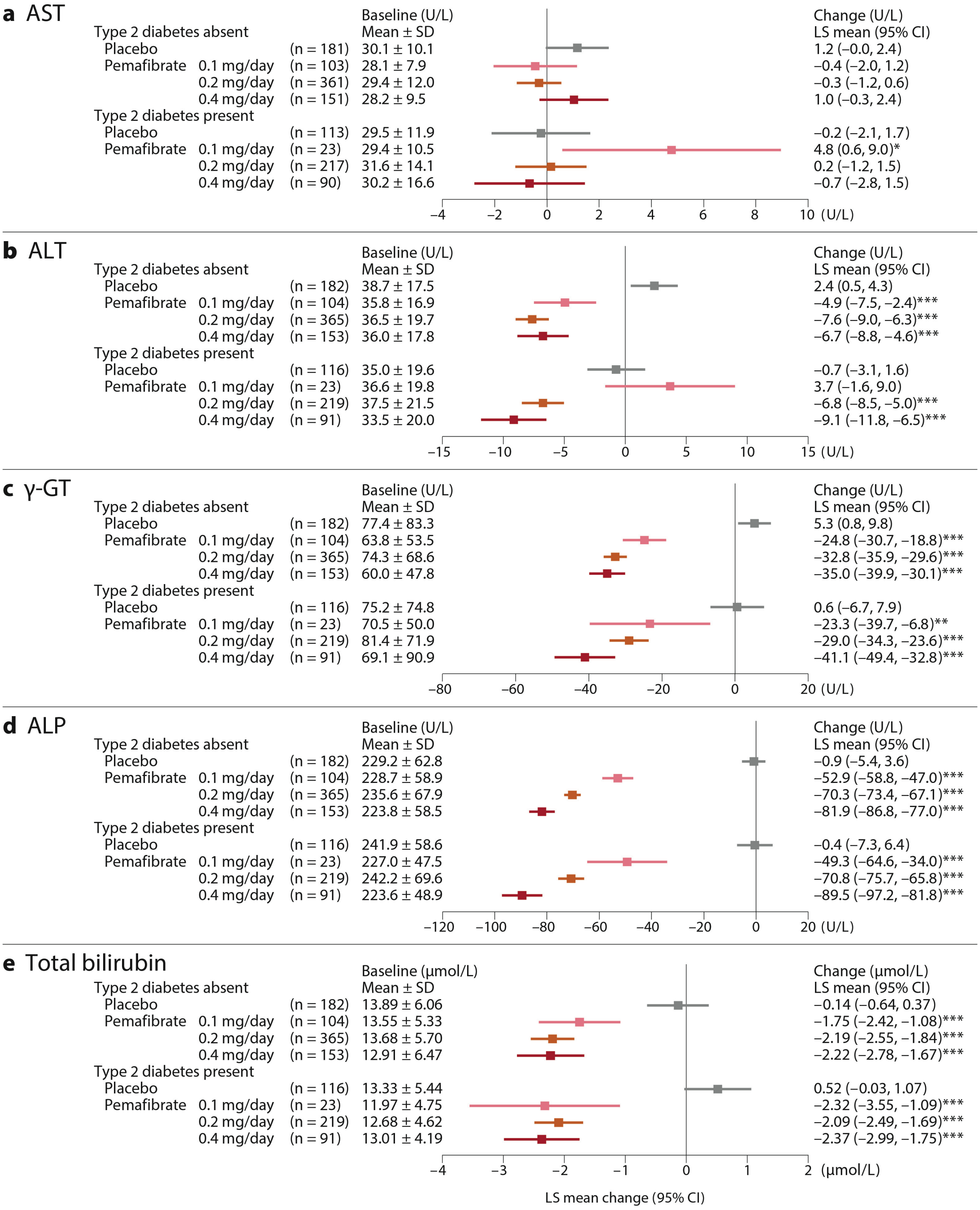

Figure S4 Changes in liver function tests, from baseline to Week 12 (LOCF), by presence or absence of type 2 diabetes. * $p < 0.05$, ** $p < 0.01$, *** $p < 0.001$ vs. placebo (ANCOVA with baseline value as covariate). *AST* aspartate aminotransferase, *ALT* alanine aminotransferase, *γ-GT* γ-glutamyl transferase, *ALP* alkaline phosphatase, *SD* standard deviation, *LS* least squares, *CI* confidence interval, *LOCF* last observation carried forward, *ANCOVA* analysis of covariance

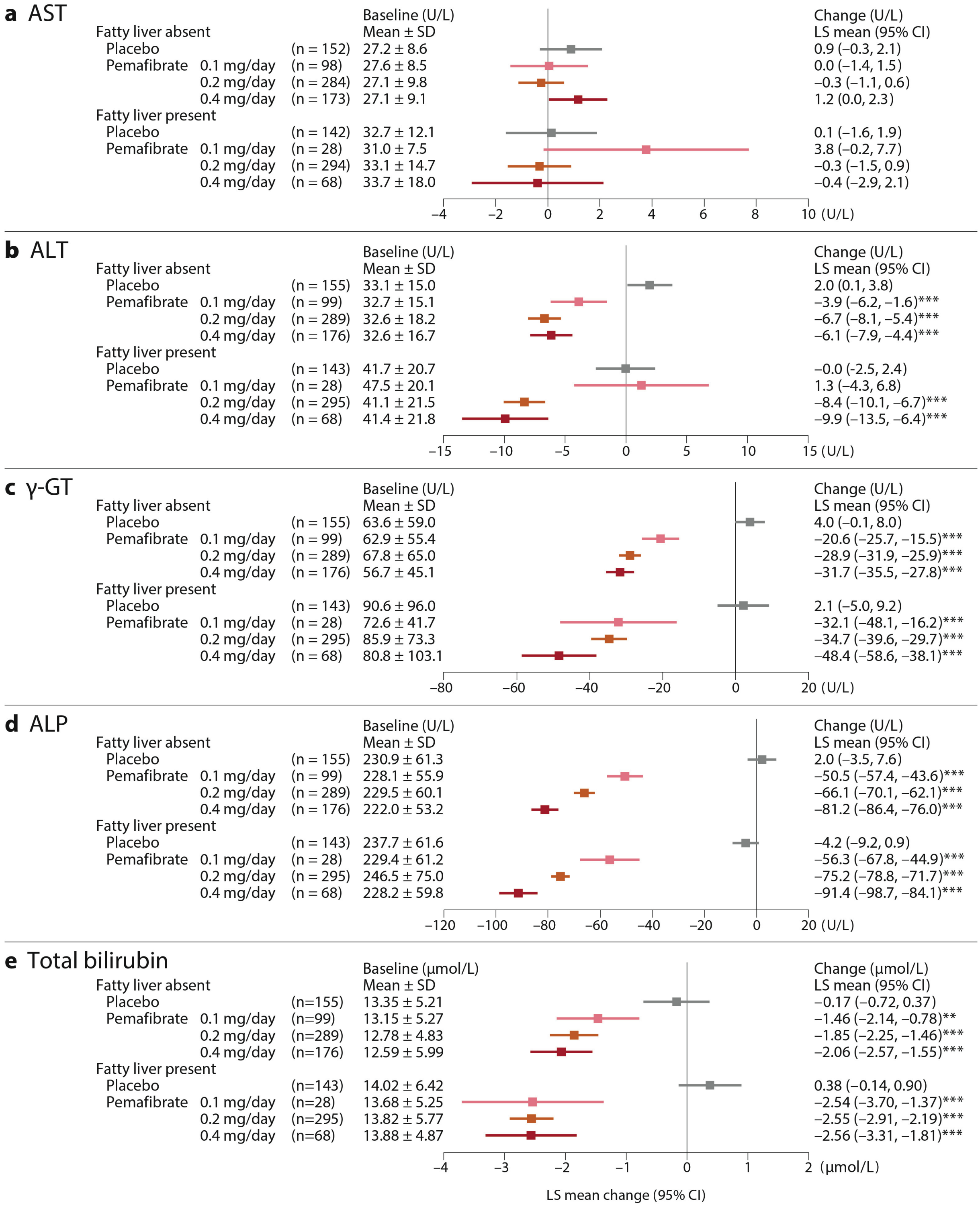

Figure S5 Changes in liver function tests, from baseline to Week 12 (LOCF), by presence or absence of fatty liver. * $p < 0.05$, ** $p < 0.01$, *** $p < 0.001$ vs. placebo (ANCOVA with baseline value as covariate). *AST* aspartate aminotransferase, *ALT* alanine aminotransferase, *γ-GT* γ-glutamyl transferase, *ALP* alkaline phosphatase, *SD* standard deviation, *LS* least squares, *CI* confidence interval, *LOCF* last observation carried forward, *ANCOVA* analysis of covariance
